# *Dead ringer* acts as a major regulator of juvenile hormone biosynthesis in insects

**DOI:** 10.1093/pnasnexus/pgae435

**Published:** 2024-09-30

**Authors:** Takumi Kayukawa, Keisuke Nagamine, Tomohiro Inui, Kakeru Yokoi, Isao Kobayashi, Hajime Nakao, Yukio Ishikawa, Takashi Matsuo

**Affiliations:** Division of Insect Advanced Technology, Institute of Agrobiological Sciences, National Agriculture and Food Research Organization, Tsukuba, Ibaraki 305-8634, Japan; Division of Insect Advanced Technology, Institute of Agrobiological Sciences, National Agriculture and Food Research Organization, Tsukuba, Ibaraki 305-8634, Japan; Division of Insect Advanced Technology, Institute of Agrobiological Sciences, National Agriculture and Food Research Organization, Tsukuba, Ibaraki 305-8634, Japan; Division of Insect Advanced Technology, Institute of Agrobiological Sciences, National Agriculture and Food Research Organization, Tsukuba, Ibaraki 305-8634, Japan; Division of Insect Advanced Technology, Institute of Agrobiological Sciences, National Agriculture and Food Research Organization, Tsukuba, Ibaraki 305-8634, Japan; Division of Insect Advanced Technology, Institute of Agrobiological Sciences, National Agriculture and Food Research Organization, Tsukuba, Ibaraki 305-8634, Japan; Faculty of Agriculture, Setsunan University, Hirakata, Osaka 573-0101, Japan; Department of Agricultural and Environmental Biology, Laboratory of Applied Entomology, The University of Tokyo, Tokyo 113-8657, Japan; Department of Agricultural and Environmental Biology, Laboratory of Applied Entomology, The University of Tokyo, Tokyo 113-8657, Japan

**Keywords:** Dead ringer, juvenile hormone, biosynthesis, metamorphosis, insect development

## Abstract

In holometabolous insects, proper control of the production of juvenile hormone (JH), which maintains larval traits, is crucial for successful metamorphosis. JH is produced specifically in the corpora allata (CA) via the functioning of a set of JH biosynthetic enzymes (JHBEs). Expression of JHBE genes in the CA is coordinated except for JH acid methyltransferase (*JHAMT*), which functions in the last step of JH biosynthesis. Here, we sought to determine the mechanism that enables this coordinated expression, assuming the presence of a central regulator of JHBE genes. Comparison of transcriptomes in the CA during active and inactive stages revealed the presence of 3 transcription factors, whose expression patterns matched those of JHBE genes. We propose that one of these, Dead ringer (*Dri*), is the central up-regulator of CA-specific JHBE genes including *JHAMT*, based on the following findings: (ⅰ) Knockdown of *Dri* in the larvae caused precocious metamorphosis, which was rescued by the exogenous application of JH analog, and (ⅱ) knockdown of *Dri* decreased the expression of most CA-specific JHBE genes examined. Furthermore, RNAi-based reverse genetics indicated that *Dri* works most upstream in the control of CA-specific JHBE genes, and that shutdown of *JHAMT*, which occurs independent of other JHBE genes prior to the onset of metamorphosis, can be hypothetically explained by the presence of an unidentified repressor. Our study suggests that *Dri*, which has been known to regulate embryonic development in a wide range of animals, is conferred a new role in holometabolous insects, i.e. central regulation of CA-specific JHBE genes.

**Significance Statement**

In holometabolous insects, juvenile hormone (JH), which is synthesized specifically in the corpora allata (CA), regulates metamorphosis by preventing its precocious occurrence. Thus, proper control of JH production is crucial for normal metamorphosis. Although most JH biosynthetic enzymes (JHBEs) have been clarified, the mechanism that regulates the coordinated expression of these enzymes remained unresolved. Here, we demonstrated that Dead ringer (*Dri*), a transcription factor, is the central regulator of JH biosynthesis in the CA, up-regulating the expression of JHBE genes. This study suggests that *Dri*, which has been known to regulate embryonic development in a wide range of animals, is conferred a new role in the control of insect metamorphosis.

## Introduction

Holometabolous insects undergo complete metamorphosis, passing through the egg, larval, pupal, and adult stages. They are extremely successful animals, representing more than 60% of all described animal species on Earth. Juvenile hormone (JH) is known as a “status quo” hormone and plays a crucial role in maintaining larval characteristics by repressing precocious larval–pupal metamorphosis until sufficient growth has occurred (1). JH is a universal and unique hormone in insects that contributes significantly to their success; hence, it is gaining interest for both academic research and pest management applications (2–4). JH is specifically synthesized in the corpora allata (CA), a very small pair of glands connected to the insect brain (1). Removal of the CA from larvae induces precocious metamorphosis through the deprivation of JH (5). Conversely, in many insects, the topical application of JH analogs during larval development inhibits normal metamorphosis, resulting in supernumerary larval molts or prepupal arrest (6, 7).

**Fig. 1.**
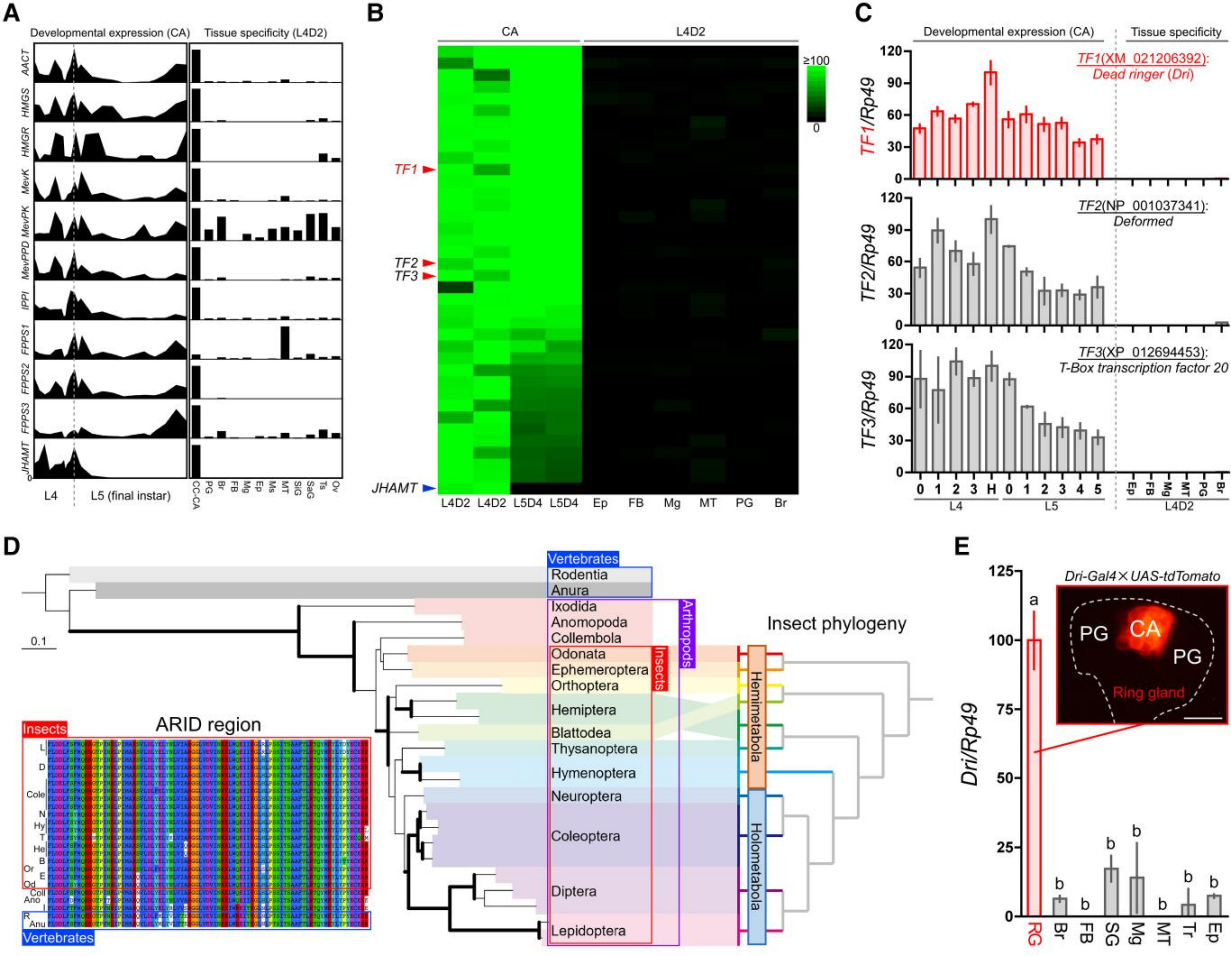
Searching for candidate central JHBE regulator genes using RNA-Seq analysis. A) Schematic of the JHBE gene expression profiles. The changes in JHBE transcripts are based on the data obtained from the previous study (11). *AACT*, *acetoacetyl-CoA thiolase*; *HMGS*, *HMG-CoA synthase*; *HMGR*, *HMG-CoA reductase*; *MevK*, *mevalonate kinase*; *MevPK*, *phosphomevalonate kinase*; *MevPPD*, *diphosphomevalonate decarboxylase*; *IPPI*, *isopentenyl pyrophosphate isomerase*; *FPPS1*, *farnesyl pyrophosphate synthase 1*; *FPPS2*, *farnesyl pyrophosphate synthase 2*; *FPPS3*, *farnesyl pyrophosphate synthase 3*; *JHAMT*, *juvenile hormone acid methyltransferase*. CC-CA, corpora cardiaca–corpora allata complex; PG, prothoracic gland; Br, brain; FB, fat body; Mg, midgut; Ep, epidermis; Ms, muscle; MT, Malpighian tubules; SiG, silk gland; SaG, salivary gland; Ts, testis; Ov, ovary. B) Heat map showing the CA-specific genes obtained from RNA-Seq analysis. The average tag count in the L4D2-CA samples of each contig was set as 100. The red arrowhead indicates transcription factor genes (*TF1–3*), and the blue arrowhead indicates *JHAMT*, the positive control. C) Developmental expression profiles and tissue specificities of *TF1–3* were determined using qPCR (*n* = 3). The samples of the CA, PG, and Br were prepared from 5 animals, and those of other tissues were prepared from 3 animals. The transcript levels of the CA on head capsule slippage (H) of the 4th instar were set as 100. The gene names and accession numbers of *TF1–3* are presented in each figure. D) Phylogenomic tree of putative Dri orthologs in insects and vertebrates (*Mus musculus* and *Xenopus tropicalis*, outgroup). The phylogeny was reconstructed using the maximum likelihood method, and the insect phylogeny is based on the data obtained from previous studies (12). Bold branch shows a bootstrap percentage that is greater than 70% support. Scale bars indicate the amino acid substitutions per site. (Inset) Alignment of the predicted amino acid sequences of ARID in insects and vertebrates. Residues are color coded according to their conservancy. The regions of ARID follow the data obtained from previous studies (13). The abbreviations on the left indicate the animal orders, corresponding to those in the phylogenomic tree. An enlarged view is shown in SI Appendix, Fig. S2. E) Tissue specificities of the *Dri* transcript in the wandering larvae (W0-6h) of *D. melanogaster* were investigated using qPCR. The transcript levels of the RG were set as 100. Data represent means ± SD (*n* = 3). Different letters indicate a significant difference among the 8 tissues (one-way ANOVA with Tukey's post hoc test; *P* < 0.05). Br, brain; SG, salivary gland; Mg, midgut; MT, Malpighian tubules; Tr, trachea; Ep, epidermis. (Inset) Localization of tdTomato (red fluorescence) driven by *Dri*-*Gal4* in the RG of the wandering larva of *D. melanogaster*. The RG is outlined by the dashed line. Scale bar, 25 μm.

The JH biosynthetic pathway in the CA is conventionally divided into early and late steps. The early step involves the classical mevalonate pathway, which is common in vertebrates and invertebrates, whereas the late step is unique to insects and other arthropods (8, 9). Most of the genes that encode JH biosynthetic enzymes (JHBEs), except for a few in the late step, have been identified in the silkworm, *Bombyx mori* (Kinsyu × Showa strain), and tissue specificity and developmental expression profiles of JHBE genes in this species have been investigated (10, 11). The JHBE genes are constitutively and specifically expressed in the CA, with some fluctuations during the larval stage [See Fig. 1A, which is prepared based on the data obtained from the previous study (11)]. However, JH acid methyltransferase (*JHAMT*), the rate-limiting enzyme that works at the final step of the JH biosynthesis pathway, is completely shut down in the final instar larva, independent of other JHBEs (Fig. 1A). Consequently, JH disappears from the hemolymph of the final instar larva, and larval–pupal metamorphosis is induced. Similar fluctuations in the expression of JHBE genes have been reported for a different strain of *B. mori*, Dazao (14).

The molecular regulation of JH biosynthesis in the CA remains unclear; particularly, the transcription factors (TFs) that regulate the expression of JHBE genes are poorly understood. Several TFs are known to partially regulate JHBE expression. A participant in Decapentaplegic (Dpp)-mediated TGF-β signaling, Mothers against dpp (Mad), has been reported to regulate JH biosynthesis via *JHAMT* expression in the CA of the fruit fly (*Drosophila melanogaster*) and a cricket (*Gryllus bimaculatus*) (15, 16). Ventral veins lacking (Vvl)/Drifter, a POU domain TF, up-regulate the *JHAMT* transcription in the red flour beetle (*Tribolium castaneum*) and *B. mori* (14, 17). In the German cockroach (*Blattella germanica*), Seven-up and FTZ-F1 drive the expression of 2 JHBEs (*HMGS* and *HMGR*) in the CA to control JH production in adulthood (18). Although these studies revealed that TFs partially regulate a few JHBE transcripts, the TF that induces the CA-specific constitutive expression of most JHBE genes is unknown.

In this study, we identified a TF gene, Dead ringer (*Dri*), as the central regulator of most JHBE genes. *Dri* is an AT-rich interaction domain (ARID) family TF, and the orthologs of *Dri*, i.e. *Dril1*, *Bright*, and *ARID3a*, are widely conserved among animals (19, 20). In vertebrates, mice (*Mus musculus*) lacking *Bright/ARID3a* suffered embryonic lethality because *Bright/ARID3a* is crucial for embryonic hematopoiesis and stem cell differentiation (21–25). Moreover, the western clawed frog (*Xenopus tropicalis*) treated with *Dril1*-morpholino showed a gastrulation defect (19). In insects, studies on *Dri* have only been reported for *D. melanogaster*, and they focused primarily on the embryonic development and its protein structure (13, 26–32). Although the orthologs of *Dri* are widely conserved among animals, the functions of *Dri*, except embryogenesis, are largely unknown, and only a few animal species have been used in *Dri* studies. Therefore, we performed a functional analysis of *Dri* in the larval stage of insects and uncovered a novel function of *Dri* in the regulation of insect development.

## Results

### Searching for a candidate JHBE regulator

The expression of JHBE genes examined to date in *B. mori* (Kinsyu × Showa strain, the strain also used in the present study) was CA-specific, and their expression patterns were largely similar except for *JHAMT* (Fig. 1A). *JHAMT* is specifically shutdown prior to the onset of metamorphosis, wherein other JHBE genes remained active (Fig. 1A). The similarity in the expression patterns of most JHBE genes led us to hypothesize the presence of a central regulator for their expression in the CA. RNA-seq analysis was performed to explore the candidates. We used 2 stages of the CA in *B. mori*, i.e. day 2 of the 4th instar larvae (L4D2), wherein JH synthesis is active, and day 4 of the 5th (final) instar larvae (L5D4), wherein JH synthesis is inactive, and 6 other tissues of L4D2. *JHAMT*, which was used as a control in this study, was highly expressed in the CA of L4D2 but was undetectable in the CA of L5D4 and the other tissues (Fig. 1B); this is consistent with previous studies (Fig. 1A). Genes that were highly expressed in the CA at both stages and barely expressed in other tissues resulted in the selection of 37 genes (Fig. 1B). The selection of *TFs* from the 37 genes narrowed the candidates down to three genes (*TF1–3*, Fig. 1B). When the expression levels of the 3 selected genes were re-measured via quantitative PCR (qPCR), they were confirmed to be expressed specifically in the CA throughout the 4- and 5th-instar larval stages examined in this study (Fig. 1C). A BLAST search of NCBI database revealed that *TF1* (XP_021206392), *TF2* (NP_001037341), and *TF3* (XM_012694453) are the orthologs of *Dri* (also known as “retained [*retn*]” in *D. melanogaster*) (29), Deformed (*Dfd*), and T-Box transcription factor 20 (*TBX20*), respectively (Fig. 1C). Of these *TFs*, we focused on the *Dri* gene and analyzed its function in JH biosynthesis, because preliminary RNAi screening using *T. castaneum* showed that knockdown of *Dri*, not the other 2, induced precocious metamorphosis (SI Appendix, Fig. S1).

Phylogenetic analysis revealed that Dri orthologs are widely distributed among insects and that their phylogeny reflects their taxonomic relationships (12) (Fig. 1D). ARIDs of Dri are highly conserved from vertebrates to insects (ARID alignment, Fig 1D inset; whole alignment, SI Appendix, Fig. S2); however, several regions that are highly conserved only in insects are observed downstream of the ARID region (SI Appendix, Fig. S2).

The tissue specificity of *Dri* expression in *D. melanogaster* was investigated in addition to that in *B. mori*. qPCR analysis of the early wandering larvae (W0–6h) revealed high levels of the *Dri* transcript in the ring gland (RG) of *D. melanogaster*, which comprises the CA and prothoracic gland (PG) (Fig. 1E). To determine whether *Dri* is expressed in the CA or PG, we used an exon trap line of *Dri*. Fluorescence observation of larvae in *Dri*-*Gal4*/*UAS*-*Tomato* showed that the *Dri* transcript was specifically expressed in the CA (Fig. 1E inset). CA-specific expression of *Dri* in 2 distantly related insects, *B. mori* and *D. melanogaster* (Fig. 1C and E), along with the high conservation of *Dri* among wide range of insects (Fig. 1D), suggests that the function of *Dri* is likely conserved in many insects.

### Functional analysis of *Dri* using genome editing in *B. mori*

**Fig. 2.**
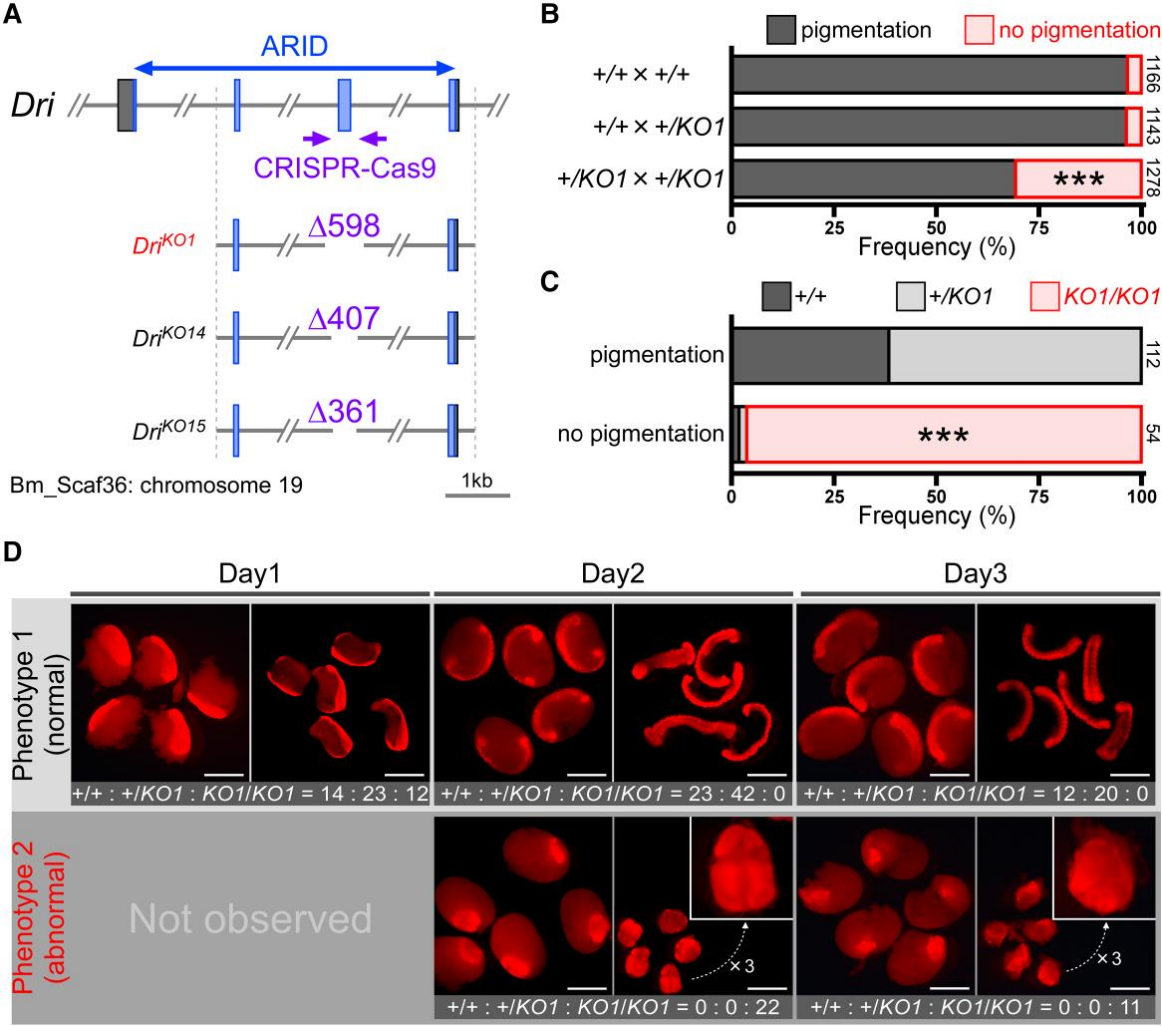
Characterization analysis of *Dri* using genome editing. A) CRISPR-Cas9 target sites in *Dri* of *Bombyx mori*. The 5th exon, encoding part of ARID, was deleted from the genome of the *pnd w-1* strain. *Dri^KO1^*, *Dri^KO14^*, and *Dri^KO15^* alleles were excised with 598, 407, and 361 bp, respectively. B) Incidences of egg phenotype. Eggs were obtained from the crosses of *+/+* × *+/+*, *+/+* × *+/KO1*, or *+/KO1* × *+/KO1* in the *Dri^KO1^* allele. The data represent the frequency (%, *n* = 1,143 to 1,278 eggs). Statistical analysis was carried out using Fisher's exact test with the raw data, with Holm-adjusted by *P* values (***P < 0.001). C) Genotype frequency (%, *n* = 54 or 112 eggs) of 2 phenotypes obtained from the sibling cross of *+/KO1*. Genotypes were determined according to SI Appendix, Fig. S3E. The data analysis was the same as B). D) Phenotypes of *Dri* null mutant embryos. Eggs derived from the sibling cross of *+/KO1* were reared at 25 °C, and the embryos were stained with propidium iodide on days 1 to 3. The left photographs represent the embryos with yolks, and those on the right represent the embryos whose yolks had been removed. Genotypes were determined using PCR after fluorescence observation. Scale bar, 1 mm.

The genome database of *B. mori* (33) revealed that *Dri* comprises 10 exons and that ARID, which recognizes AT-rich DNA sequences (13, 27), is encoded in exons 3 to 6 (SI Appendix, Fig. S3A). CRISPR-Cas9 targeting exon 5 of *Dri* yielded 17 mutant alleles, and we selected alleles *KO1*, *KO14*, and *KO15* with deletions of 598, 407, and 361 bp, respectively (Fig. 2A and SI Appendix, Fig. S3B). These knockout alleles completely lacked exon 5, representing approximately half of the ARID that is essential for their function, suggesting that they should be null alleles in the *Dri* gene (Fig. 2A). Egg batches with *Dri^KO1^*-, *Dri^KO14^*-, *Dri^KO15^*-homozygous alleles were obtained via sibling crosses of each heterozygous male and female adults. Normally, intact eggs of the wild type become pigmented 3–4 days before hatching, whereas unfertilized and undeveloped eggs do not become pigmented. Observation of the eggs revealed 2 phenotypes: pigmented and unpigmented eggs (SI Appendix, Fig. S3C). The incidence of unpigmented eggs increased significantly in the batches obtained from the *Dri^KO1^*-heterozygous crosses (approximately 30.7%), whereas no change was observed in the *Dri^+/+^* crosses or *Dri^+/+^* and *Dri^+/KO1^* crosses (approximately 3.3–3.6%) (Fig. 2B). As *Dri^KO14^*- and *Dri^KO15^*-heterozygous crosses showed phenotypes similar to *Dri^KO1^* (SI Appendix, Fig. S3D), subsequent analyses were conducted using the *Dri^KO1^* line.

The genotyping of the phenotypes of *Dri^KO1^* alleles revealed that the *Dri* null mutant (*Dri^KO1/KO1^*) showed no pigmentation and caused embryonic lethality in *B. mori* (Fig. 2C and SI Appendix, Fig. S3E). To investigate the embryonic morphology of the *Dri* null mutant in detail, we dissected the eggs and stained the embryos of the *Dri^KO1^*-heterozygous cross with propidium iodide. On day 1, only normal morphology was observed (starting gastrulation and segmentation of the germ band) (34), and the genotypes (*Dri^+/+^*:*Dri^+/KO1^*:*Dri^KO1/KO1^* = 14:23:12) nearly followed a 1:2:1 segregation via Mendelian inheritance (Fig. 2D). Two embryonic phenotypes appeared on day 2: one was a normal and elongated embryo (34), and the other was an abnormal embryo, in which the cells did not exhibit normal embryonic morphology and were clumped together (Fig. 2D). All genotypes in the abnormal embryo indicated *Dri*-knockout homozygous alleles (*Dri^+/+^*:*Dri^+/KO1^*:*Dri^KO1/KO1^* = 0:0:22), whereas those of the normal embryos were *Dri^+/+^*:*Dri^+/KO1^*:*Dri^KO1/KO1^* = 23:42:0. On day 3, the normal embryos developed as expected: shortened embryos with formed appendages were observed (34), whereas abnormal embryos remained aggregated (Fig. 2D). Therefore, the *Dri* null mutant caused abnormal embryogenesis between days 1 and 2, leading to embryonic lethality. To examine whether this abnormal embryogenesis was attributed to JH deficiency caused by the knockout of *Dri*, a JH analog (JHA, methoprene) was topically applied to the eggs on either day 0 or 1 after oviposition. Only the 2 phenotypes shown in Fig. 2D were observed. The genotypes of normal embryos were *Dri^+/+^*:*Dri^+/KO1^*:*Dri^KO1/KO1^* = 8:24:0 and 13:19:0, respectively (SI Appendix, Table S1), suggesting that JHA did not rescue the abnormal embryogenesis. This finding indicates that *Dri* fulfills a crucial function during early embryogenesis in *B. mori*.

### *Dri* regulates JHBE gene expression during the larval stage

Loss-of-function analysis of *Dri* in the larval stage of *B. mori* was difficult, because its *Dri* null mutant exhibited embryonic lethality (Fig. 2) and the efficiency of RNAi in lepidopteran species are disputable (35, 36). JH is a ubiquitous hormone and its biosynthetic and signaling pathways are widely conserved in insects (1, 8, 9, 37–39). Therefore, we performed further analyses using a coleopteran insect, the yellow-spotted longicorn beetle (*Psacothea hilaris*), to characterize the role of *Dri* in JH biosynthesis during the larval stage. This species is suitable for this study because its RNAi experiments are effective and the CA can be easily dissected from the larva, which is several times larger than that of the model coleopteran insect, *T. castaneum*.

**Fig. 3.**
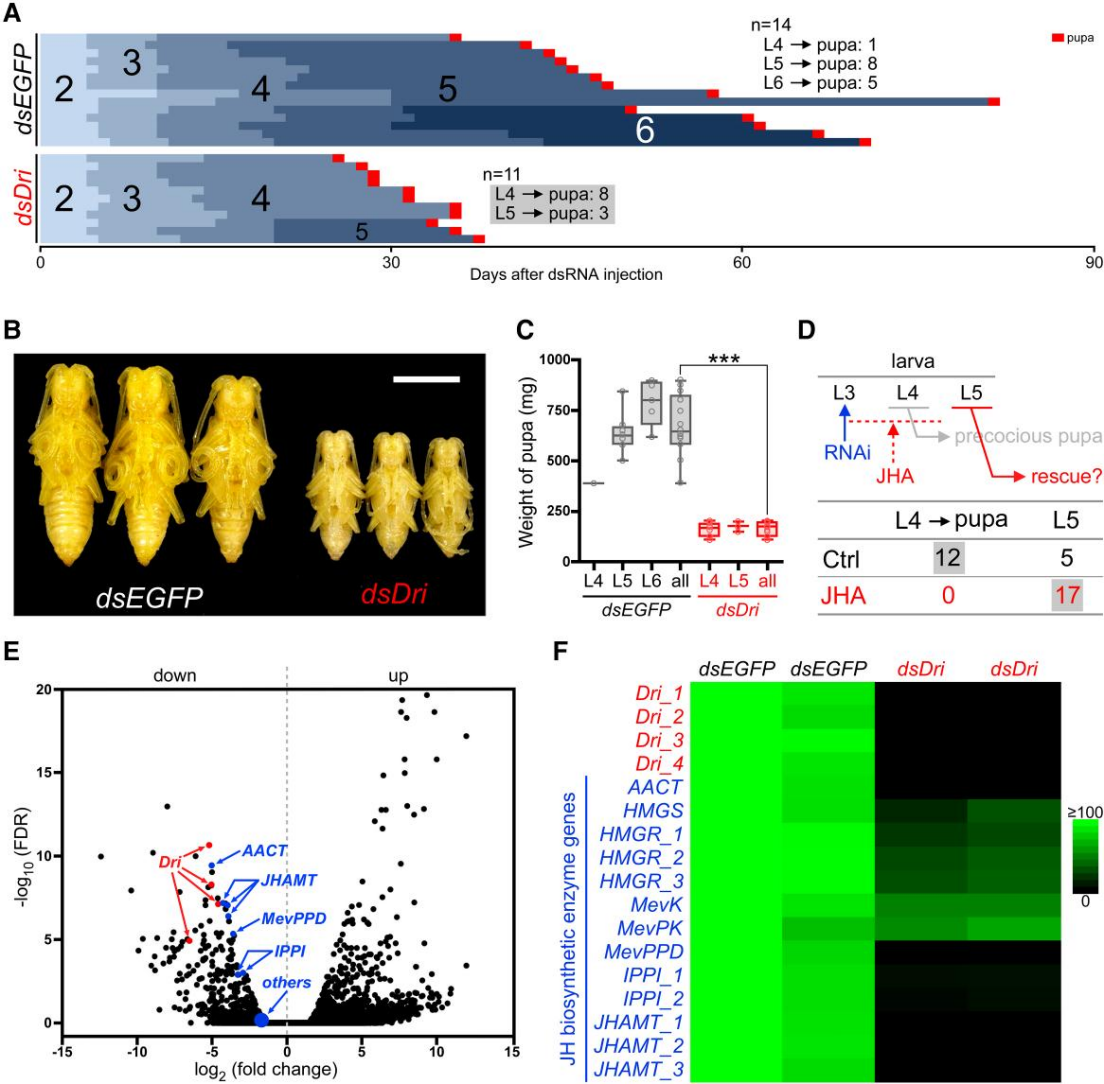
*Dri* regulates the expression of JHBE genes in the CA. A) dsRNAs of *EGFP* (negative control) or *Dri* was injected into *P. hilaris* on L2D0, and the occurrence of precocious metamorphosis was observed (*n* = 11 or 14). The numbers represent the larval instar. B) Phenotypes of *P. hilaris* obtained from A). *dsEGFP* shows the pupae metamorphosed from 5th instar larvae, and *Dri-*RNAi pupae precociously metamorphosed from 4th instar larvae. Scale bar, 1 cm. C) Weights of fresh pupae obtained from individuals represented in A). “All” indicates that the various instar pupae in each dsRNA treatment were pooled and the data analyzed using Student's *t*-tests (***P < 0.001). D) Rescue experiment with JHA. JHA solution was topically applied to the dorsal abdomen of the larvae immediately after *dsDri* injection on L3D0. The topical application was continued every 3 days and immediately after molting. The inhibition of precocious metamorphosis was evaluated. Ctrl, acetone alone; JHA, methoprene. E) Volcano plot shows the results of Smart-seq using the CA samples (*n* = 2) after the injection of *dsEGFP* or *dsDri*. Red and blue dots indicate the *Dri* and JHBE gene contigs, respectively. F) Heat map of *Dri* and JHBE contigs extracted from the volcano plot of E). The average tag count in *dsEGFP* samples of each contig was set as 100.

The L2D0 *P. hilaris* larvae injected with *dsEGFP* (negative control) metamorphosed to pupae primarily during the 5 or 6th instar (Fig. 3A). In contrast, *dsDri* injection led to precocious metamorphosis during the 4th instar (Fig. 3A) as did *dsJHAMT* (SI Appendix, Fig. S4A), resulting in miniature pupae (Fig. 3B). The body weight of pupae in the *Dri*-RNAi treatment, which were derived from different instars, was not significantly different, and the weight after *Dri*-RNAi treatment decreased by approximately a quarter compared with that after *EGFP*-RNAi treatment (Fig. 3C). To examine whether the precocious metamorphosis was attributed to JH deficiency caused by *Dri* RNAi, JHA was topically applied to the larvae after the injection of *dsDri* at L3D0 (Fig. 3D). This treatment would not be able to prevent precocious metamorphosis if *Dri* is not involved in JH biosynthesis but in JH signaling in target cells or performing other functions. Topical application of JHA prevented *Dri*-RNAi larvae from metamorphosing precociously during the 4th instar stage, and all the larvae molted to the 5th instar (Fig. 3D). The size of the CA in *P. hilaris* larvae was not affected by *Dri*-RNAi treatment (SI Appendix, Fig. S4B). In *T. castaneum*, *Dri* RNAi caused precocious metamorphosis from the 6th instar, whereas a negative control (*dsMalE*) showed pupation from the 7 or 8th instar (SI Appendix, Fig. S5A). The precocious pupae were miniature in size compared with those formed from the 7th instar larvae treated with *dsMalE* (SI Appendix, Fig. S5B), and precocious metamorphosis was prevented by JHA (SI Appendix, Fig. S5C).

To further clarify the function of *Dri*, we carried out Smart-seq, a single-cell mRNA sequencing protocol (40), using the CA samples of *P. hilaris* 4 days after *Dri* RNAi was injected on L3D0. The volcano plot showed that after *Dri*-RNAi treatment, the expression levels of 4 *Dri* contigs were significantly reduced, and the levels of several *JHBE* contigs also declined (Fig. 3E). Therefore, we generated heat maps for the JHBE genes identified from the volcano plot (Fig. 3F). Figure 3F depicts that the transcript levels of JHBE genes, i.e. *AACT*, *HMGS*, *HMGR*, *MevK*, *MevPK*, *MevPPD*, *IPPI*, and *JHAMT*, decreased following *Dri*-RNAi treatment. These findings suggest that *Dri* regulates the expression of most JHBE genes in the CA.

### Relationship between *Dri* and other regulatory factors in JH biosynthesis

Dpp-mediated TGF-β signaling has been reported to regulate JH biosynthesis via *JHAMT* expression in the CA of *D. melanogaster* and *G. bimaculatus* (SI Appendix, Fig. S6A) (15, 16). We investigated the relationship between *Dri*, *TGF-β*, and *JHAMT* in *P. hilaris*. At the transcript level, *Dri* RNAi drastically reduced *JHAMT* expression but did not affect the expression of *Dpp* and *Mad* (SI Appendix, Fig. S6B). In contrast, no changes in the *Dri* and *JHAMT* transcript levels were observed in the CA of *Dpp-* or *Mad*-RNAi larvae, although their own transcripts were significantly suppressed by RNAi (SI Appendix, Fig. S6C and D). These results suggest that the regulation of JH biosynthesis by *Dri* is independent from TGF-β signaling in *P. hilaris*.

Although *Dri* RNAi induced precocious metamorphosis (Fig. 3A, B and SI Appendix, Fig. S5A and B), the RNAi of 2 participants in TGF-β signaling, i.e. *Dpp* and *Mad*, did not cause precocious metamorphosis but did cause larval arrest at the 3rd or 4th instar (SI Appendix, Fig. S6E). These RNAi treatments induced translucent larvae (SI Appendix, Fig. S6F), which shriveled and died within a few months. Microscopic observation of the dissected larvae showed that the fat body was barely observed in the *Mad*-RNAi larvae compared with that in the *EGFP*-RNAi control larvae (SI Appendix, Fig. S6G).

**Fig. 4.**
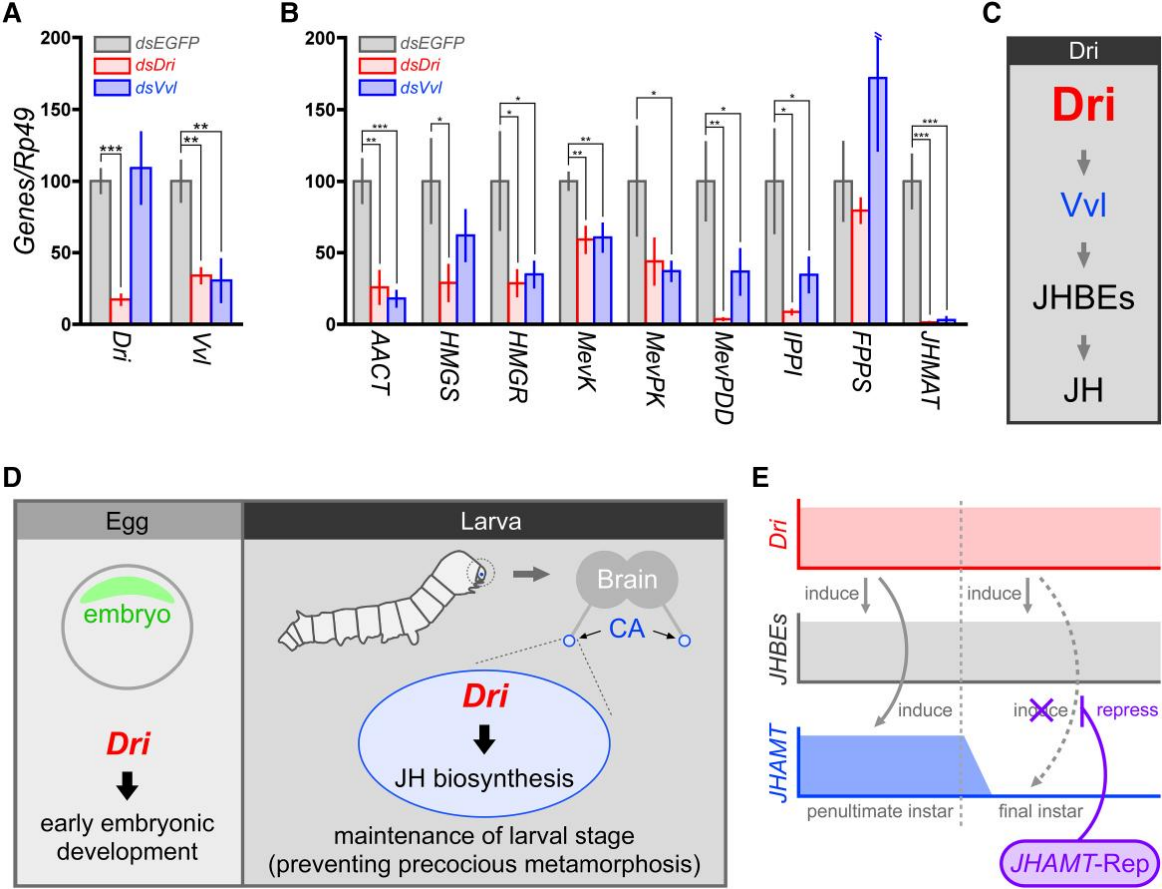
Relationship between *Dri* and *Vvl* in JH biosynthesis. A) Expression levels of *Dri* and *Vvl* were examined in the CA derived from *P. hilaris* larvae on day 4 after injection with *dsEGFP* (negative control), *dsDri*, or *dsVvl* on L3D0. The data represent the means ± SD (*n* = 3–5) and were analyzed using Student's *t*-tests (***P < 0.001; **P < 0.01; *P < 0.05; not indicated, *P* > 0.05). B) Expression levels of JHBE genes were examined in the CA derived from the larvae on day 4 after injection with *dsEGFP*, *Dri*, or *Vvl* on L3D0. The samples and data analysis are the same as A). C) A model for the regulatory pathway of JH biosynthesis by *Dri* in the CA. D) Summary model for the role of *Dri* in insect development. During the early embryonic stage, *Dri* fulfills a crucial function in embryonic development, which is independent of JH biosynthesis. In contrast, during the larval stage, *Dri* functions as a central regulator of JH biosynthesis, thereby maintaining the larval form. E) A regulation model of JH biosynthesis in metamorphosis. *Dri* up-regulates JHBE genes, including *JHAMT*, throughout the larval stage. In the last instar, a *JHAMT*-specific repressor (*JHAMT*-Rep), which counteracts the up-regulation by *Dri*, specifically represses the *JHAMT* transcription and shuts down JH biosynthesis, thereby inducing larval–pupal metamorphosis.

*Vvl*, a POU domain TF, has also been shown to regulate JH biosynthesis via the up-regulation of the *JHAMT* transcript in *T. castaneum* and *B. mori* (14, 17). The roles of *Dri* and *Vvl* in JH biosynthesis were examined by injecting dsRNA into the larvae of *P. hilaris*. We examined the expression levels of *Dri* and *Vvl* using a sample derived from the CA of *P. hilaris* 4 days after dsRNA treatment at L3D0. *Dri* RNAi significantly reduced the expression of *Vvl*, whereas *Vvl* RNAi did not reduce *Dri* expression (Fig. 4A).

As shown in Fig. 3F, *Dri* regulated the expression of several JHBE genes in the CA. Furthermore, a recent study using *B. mori* showed that *POU-M2*, an ortholog of *Vvl*, promotes JH biosynthesis by directly activating the transcription of various JHBE genes (14). Thus, we investigated the expression of JHBE genes, which are listed in Fig. 1A, using the same CA samples. Overall, changes in the expression of JHBE genes were similar in *Dri*- and *Vvl*-RNAi, and significant decreases in the transcript levels of JHBE genes, except for *FPPS*, were observed (Fig. 4B). These findings indicate that *Dri* up-regulates the CA-specific expression of most JHBE genes upstream of *Vvl* in the CA (Fig. 4C).

## Discussion

The larval stage of insects is devoted to feeding and growth until they reach a sufficient size for adulthood. JH is the key hormone responsible for maintaining larval characteristics (1). Elucidating JH biosynthesis at the molecular level is a challenge in insect physiology and developmental biology. The present study identified *Dri* as a novel central regulator of JH biosynthesis and clarified the molecular mechanisms underlying its regulation of JHBE genes.

We generated genome-edited silkworms with knocked-out *Dri* to investigate the function of *Dri* in *B. mori*. If *Dri* regulates JH biosynthesis in *B. mori*, then the *Dri* null mutant would show precocious metamorphosis. Typically, *B. mori pnd w-1* requires approximately 12 days at 25 °C for embryonic development till hatching. However, the *Dri* null mutant exhibited an abnormal phenotype at the early embryonic stage: the germ band agglomerated between days 1 and 2, and this was not rescued by JHA. Moreover, based on the JH titer during the embryogenesis of the tobacco hornworm (*Manduca sexta*) (41) and the *JHAMT* expression during embryogenesis in *B. mori* (42), the JH titer in the embryos of *B. mori* is presumed to increase from day 4 (42). These results indicate that *Dri* plays an important role in early embryonic development but not in JH biosynthesis during the early embryonic stage (Fig. 4D). In insects, the phenotype of the *Dri* null mutant during early embryogenesis has only been reported in *D. melanogaster* (26, 28, 31). In vertebrates, moreover, loss-of-function analysis of *Dri* orthologs displayed early embryonic lethality and a gastrulation defect (19, 21, 22). These results suggest that *Dri* has crucial functions in embryogenesis, which are widely conserved in animals.

In *D. melanogaster*, *Dri* plays an essential role in early anterior–posterior patterning and muscle development during embryogenesis; its mutant shows segmentation, head defects, and an ectopic cephalic furrow (28). Moreover, *Dri* has been reported to participate in dorsal–ventral axis formation in the early embryo (26), suggesting that *Dri* functions in multiple separate processes during embryonic development. Notably, *Dri* preferentially binds to the A/GATTAA sequence, which is strikingly similar to that preferred by several homeodomain proteins that are essential for early embryogenesis (13). However, there have been no further reports on *Dri* in insects since 2005 (32, 43). Comprehensive gene analysis using current NGS techniques, such as RNA-seq and ChIP-seq, may provide additional insights into the *Dri*-mediated regulation of insect embryogenesis.

*Dri* is constitutively expressed, specifically in the CA of larva, throughout the active stage to complete cessation of JH biosynthesis. *Dri* RNAi induced precocious metamorphosis in *P. hilaris* and *T. castaneum*, which was rescued by JHA treatment, suggesting that *Dri* works upstream of the JH signaling pathway in the target cell. Smart-seq analyses revealed that various JHBE genes in the CA were down-regulated by *Dri* RNAi. These findings indicate that *Dri* up-regulates the constitutive expression of JHBE genes in the CA of larvae. Moreover, reverse genetics analysis using RNAi and qPCR indicated that *Dri* up-regulated the expression of JHBE genes upstream of *Vvl*. Taken together, these results show that *Dri* functions as a central regulator of JH biosynthesis during the larval stage (Fig. 4D). Phylogenetic analysis revealed that putative *Dri* orthologs are highly conserved among species across a wide range of insect orders. Moreover, CA-specific expression of *Dri* in the larvae of *B. mori* and *D. melanogaster*, two species in different orders, suggests that the central regulation of JH biosynthesis by *Dri* is conserved in a wide range of insect groups.

In holometabolous insects, JH titer is maintained at high levels until the penultimate instar and declines to trace levels in the final instar (1). As for its molecular regulation, the JHBE genes, except *JHAMT*, are constitutively expressed throughout the larval stage despite some fluctuations (10, 11). Although the present study showed that *Dri*, as with *Vvl*, up-regulates the expression of *JHAMT*, the *JHAMT* transcript level declines rapidly early in the final instar and completely disappears within a few days (10, 11). Based on these findings, we hypothesized that *Dri* and *Vvl* act as up-regulators for many JHBE genes, including *JHAMT*, throughout the larval stage; however, a *JHAMT*-specific repressor, which counteracts the up-regulation by *Dri* and *Vvl*, appears in the final instar, and hence only the *JHAMT* transcript is completely shut down (Fig. 4E). In a hemimetabolous insect (*G. bimaculatus*), JH titers exhibit periodic changes, with a peak level at the beginning of each instar, declining to a low level at the end of the stage, in accordance with the changes observed in *JHAMT* transcripts (16). In addition, myoglianin (*Myo*) transcripts show periodic changes, with a peak in the middle of each instar. Myo-mediated TGF-β signaling is involved in the down-regulation of JH biosynthesis through suppression of the expression of *JHAMT* (16). A recent study suggested that this signaling is also involved in the repression of JH biosynthesis in a coleopteran insect, *T. castaneum* (44). Further analysis of Myo-mediated TGF-β signaling in holometabolous insects is needed to understand the molecular mechanism of *JHAMT* repression.

JH has also been reported to play roles in reproductive behavior, such as the maturation of female receptivity and sex pheromone production, male mating behavior, and courtship memory in *D. melanogaster* adults (45–49). Females of *D. melanogaster* reject male courtship attempts immediately after eclosion, and most of them only become receptive and mate by day 2 (45). Implantation of the CA or application of methoprene causes females to mate precociously (45, 50), and conversely, JH receptor (*Met*) and JH deficiency (*apterous*) mutants or JH biosynthetic inhibitor (precocene) treatment reduce female mating (46, 51, 52). The missense alleles *retn^Z2–428^* (Y364N) and *retn^RU50^* (R339C) in *D. melanogaster* encode a Dri protein with sufficient function, enabling the mutant progeny to survive to adulthood, whereas the amorphic allele *retn^dri2^* is embryonically lethal (43). Experiments using heteroallelic combinations with these mutant alleles revealed that females showed striking resistance to male courtship (43). Notably, this phenotype is a phenocopy of JH deficiency, as mentioned above. If *Dri* is also involved in JH biosynthesis in the adult stage, the mating behavior defects in the *Dri* mutant may be attributed to JH deficiency.

Our study provided valuable insights into regulation of JH biosynthesis by *Dri* during the larval stages of insects. Although the importance of *Dri* in embryogenesis is common in various animals, our findings suggest that *Dri* can evolutionarily drive the diverse life-history traits of animals by regulating the biosynthesis of the hormones regulating development. Further analysis of *Dri* functions would provide a deeper understanding of the fundamental mechanisms underlying insect development, which, in turn, will provide a substantial contribution to the effective utilization of insects and pest management.

## Materials and methods

A detailed description of the Materials and methods used in this study is provided in SI Appendix, Materials and Methods.

### Statistical analyses

Sample sizes were chosen based on the number of independent experiments required for statistical significance and technical feasibility. Data on the egg phenotype and genotype of the *B. mori* mutant were subjected to the Fisher's exact test, and the *P* values were adjusted using the Holm method. Data on body weights and CA diameters of *P. hilaris* and qPCR, except for tissue specificities of the *Dri* transcript in *D. melanogaster*, were analyzed using Student's *t*-tests. Tissue specificities of the *Dri* transcript in *D. melanogaster* were analyzed using one-way ANOVA with Tukey's post hoc test. Statistical analyses were performed using R (R Core Development Team, https://www.r-project.org) and Prism software (GraphPad Software, Boston, USA). All the data and statistical results have been summarized in Dataset S1.

## Supplementary Material

pgae435_Supplementary_Data

## Data Availability

Raw data of RNA-seq and Smart-seq were deposited in the Sequence Read Archive (SRA) in DDBJ with the dataset identifier PJDB15794. Other data and statistical results have been summarized in Dataset S1.
