# Supplementary material for: Dead ringer acts as a major regulator of juvenile hormone biosynthesis in insects
Source: PNAS Nexus. 2024 Sep 30;3(10):pgae435. doi: 10.1093/pnasnexus/pgae435 (PMC11467689; doi:10.1093/pnasnexus/pgae435)
Supplement: pgae435_Supplementary_Data [file pgae435_supplementary_data.zip › Supplementary Information.docx]

**Supplementary Information for**

*Dead ringer* acts as a major regulator of juvenile hormone biosynthesis in insects

Takumi Kayukawa^a,1,^*, Keisuke Nagamine^a,1^, Tomohiro Inui^a^, Kakeru Yokoi^a^, Isao Kobayashi^a^, Hajime Nakao^a^, Yukio Ishikawa^b,c^, Takashi Matsuo^b^

^a^ Division of Insect Advanced Technology, Institute of Agrobiological Sciences, National Agriculture and Food Research Organization, Tsukuba, Ibaraki 305-8634, Japan.

^b^ Faculty of Agriculture, Setsunan University, Hirakata, Osaka 573-0101, Japan.

^c^ Laboratory of Applied Entomology, Department of Agricultural and Environmental Biology, The University of Tokyo, Tokyo 113-8657, Japan.

^1^ Takumi Kayukawa and Keisuke Nagamine contributed equally to this work.

* Corresponding author: Takumi Kayukawa.

**Email:** kayu@affrc.go.jp.

**This PDF file includes:**

Supporting text

Figure S1-6

Tables S1-2

SI References

**Supporting Information Text**

**SI Materials and Methods**

**Insects and maintenance**

Kinsyu × Showa strain of *B. mori* was purchased from Uedasansyu, Ueda, Japan. Strain *pnd* *w-1* is maintained in National Agriculture and Food Research Organization, Japan. The larvae of both strains were reared on an artificial diet (SilkMate PS, Nosan Corporation, Yokohama, Japan) at 25 °C under a 12-h light/dark cycle. *Psacothea hilaris* adults were collected in Tsukuba City and Fukuchiyama City (Japan), and the larvae were reared on an artificial diet (Insecta LFM, Nosan Corporation) at 25 °C under a 16-h light: 8-h dark cycle. A wild-type strain of *T. castaneum*, a gift from Akihiro Miyanoshita (National Agriculture and Food Research Organization, Japan), was reared on whole wheat flour at 30 °C in the dark. *Drosophila melanogaster* was reared on standard agar-cornmeal medium at 25 °C in the dark. Canton-S (the wild-type) and *UAS-tdTomato* strains were gifts from Masami Shimoda (National Agriculture and Food Research Organization, Japan) and Tetsuya Tabata (The University of Tokyo, Japan), respectively. *Dri-Gal4 exon trap* strain (*retn^1405-G4^*) was obtained from Bloomington Drosophila Stock Center (Indiana University, USA). For all experiments, both males and females were used.

**RNA-seq and Smart-seq analyses**

Larval tissues were dissected at various stages in phosphate-buffered saline (137 mM NaCl, 8 mM Na_2_HPO_4_, 2.7 mM KCl, and 1.5 mM KH_2_PO_4_, pH 7.4). CA samples of *B. mori* (Kinsyu × Showa strain) were prepared from L4D2 larvae (n = 117–120 larvae) and L5D4 larvae (n = 80–90 larvae). Samples from other tissues (epidermis, fat body, midgut, Malpighian tubules, PG, and brain) were derived from L4D2 larvae (n = 10, 10, 10, 10, 10, and 95 larvae, respectively). Total RNA was extracted from the tissues using an RNeasy Plus Mini Kit (Qiagen, Hilden, Germany). For RNA-seq analyses, total RNA was treated with a TruSeq RNA Sample Prep Kit v2 (Illumina, San Diego, USA) and sequenced by Macrogen (Seoul, South Korea) using a HiSeq 2500 Sequencing System (Illumina), yielding approximately 101-bp paired-end reads. To remove adapter sequences and low-quality regions in the raw sequence data, sequences were trimmed by Trimmomatic version 0.36 using the Illumina clip data file (1). The trimmed RNA-seq data from multiple tissues of *B. mori* were mapped to reference genome data (2) using Hisat2 version 2-2.1.0 (3), and SAM output files were converted to BAM files using SAMtools version 0.1.12a (4). The mapped data were assembled into transcripts, and the assembled data of each RNA-seq were merged with the gene dataset of the reference genome into one transcriptome dataset using StringTie version 1.3.3b (3). The merged GTF data were converted to FASTA files using gffread version 0.12.1 (https://github.com/gpertea/gffread). The fragments per kilobase of transcript per million mapped reads (FPKM) values of the transcriptomes were calculated using RSEM version 1.2.7 (5), run by “align_and_estimate_abundance.pl” in the Trinity package version Trinity r20140717 (6).

To perform a Smart-seq analysis on the CA of *P. hilaris*, the CAs from L3D4 larvae (n = 16–19 larvae) that were injected with dsRNA at L3D0 were dissected. Total RNA was extracted using the RNeasy Plus Mini Kit, treated with a Smart-seq v4 Ultra Low Input RNA Kit for Sequencing (Clontech Laboratories, Mountain View, USA), and sequenced using a NovaSeq 6000 Sequencing System (Illumina), yielding approximately 150-bp paired-end reads. The raw sequence data were loaded to Trimmomatic version 0.36 to remove the low-quality regions and adapter sequences, as described previously (1). *De novo* assembly of transcripts derived from the CA samples of *P. hilaris* was performed using Trinity version 2.4.0 (4). Transcripts per million (TPM), mapped tag counts as “expected_count” (not normalized), and FPKM number (normalized) were estimated using RSEM version 1.2.7 (5), which was operated using “align_and_estimate_abundance.pl” in Trinity r20140717 as described previously (6). Differentially expressed genes (DEG) were detected using the “expected_count” data and iDEGES/edgeR in the TCC package version 1.8.2 (a false discovery rate [FDR] < 0.05 and fold changes of normalized tag count > 2) (7).

**qPCR analysis**

Total RNA was extracted from insects and tissues using the RNeasy Plus Mini Kit and used to synthesize cDNA using the PrimeScript RT reagent Kit (Takara Bio, Kusatsu, Japan). The primers designed to quantify the transcripts are listed in *SI* *Appendix*, Table S2. *Rp49* was used as the internal reference. Reactions were performed in a 10-μL volume containing template cDNA derived from 1 ng of total RNA, 5 μL of TB Green Premix Ex Taq II (Takara Bio), and 0.2 μM of each primer. A LightCycler 96 Instrument (Roche, Basel, Switzerland) was used, and the PCR conditions were 95 °C for 5 min, followed by 55 cycles of 95 °C for 5 s and 60 °C for 20 s. Relative gene expression was determined using the 2^-ΔΔCt^ method (8).

**Phylogenetic analysis and alignment of Dri orthologs**

Dri orthologs were obtained from the NCBI RefSeq protein database using a BLASTp search with the full-length sequence of *B. mori* Dri as the query. Dri orthologs were retrieved from 21 species, including 19 insect species from 11 orders and two outgroup vertebrate species. The sequences were aligned using MAFFT v7.505 with the G-INS-i algorithm with the option --maxiterate 1000 (*SI* *Appendix*, Fig. S2) (9, 10). The alignment was trimmed using the Automated1 algorithm of the TrimAl program v1.4.15 (11) to reduce it to conserved and informative sites (the trimmed alignment in FASTA format file of Dataset S2) and then subjected to a search for the best evolutionary model using ModelTest-NG v0.1.7 (12). The phylogenetic tree was reconstructed using RAxML v8.2.12 (13) with the following parameters: raxmlHPC-PTHREADS-SSE3 with -m PROTGAMMAILGF -N autoMRE_IGN. In total, 1000 bootstrapping runs were performed to satisfy the autoMRE requirement. The tree information is available in the Newick format file of Dataset S3. The phylogenetic tree was rendered using the web server iTOL v6.7 (14).

**Genome editing**

Specific sgRNAs targeting exon 5 of *Dri* were designed using CRISPR Direct (15). Following a reported method (16), each DNA template was PCR-amplified for sgRNA synthesis using KOD Plus Neo DNA polymerase (Toyobo, Osaka, Japan) and primers. The forward primers encoded the T7 polymerase binding and sgRNA target sites, and the reverse primer was based on a common sequence encoding the remainder of the sgRNA sequence (*SI* *Appendix*, Table S2). The PCR products were purified using the Wizard SV Gel and PCR Clean-Up System (Promega, Madison, USA). The transcription of sgRNAs was performed using the MEGAshortscript T7 Transcription Kit (Thermo Fisher Scientific, Waltham, USA), in which 300 ng of the purified DNA template was incubated at 37 °C for 4 h. The concentrations of sgRNAs and Alt-R S.p. Cas9 Nuclease V3 (Integrated DNA Technologies, Coralville, USA) were adjusted to 75 and 0.75 μg/μL, respectively, using sterile water. The solution was injected into *B. mori* *pnd* *w-1* eggs at the preblastoderm stage. For genotyping, hemolymph samples were directly added to 100 mM NaOH, and the embryonic and larval samples were homogenized in 100 mM NaOH using a manual pestle. Samples were then incubated at 95 °C for 15 min. The samples were neutralized with half the volume of 1 M Tris-HCl (pH 8.0) and used as a genomic DNA template for PCR. Reactions were performed in a 20-μL volume containing the genomic DNA template and primers (*SI* *Appendix*, Table S2) using KOD FX Neo DNA polymerase (Toyobo). The PCR products were electrophoresed at 25 °C on a 1.5% agarose gel in 0.5× TBE, and the genotypes were determined based on the band patterns. *Dri*-mutant lines were maintained as heterozygous stock: the hemolymph collected from pupae was genotyped to obtain egg batches from sibling crosses of heterozygous male and female adults.

**Embryo staining**

The embryos were placed in PBS, and a part of the chorion was carefully removed using a surgical knife. Subsequently, the embryos were fixed overnight in 4% formaldehyde in PBS. The fixed embryos were washed three times in PBS containing 0.1% Triton-X-100 (PBST) and stained overnight with propidium iodide (PI, DOJINDO Laboratories, Kumamoto, Japan) in PBST at 4 °C. After washing with PBST, the stained embryos with yolk were observed using a motorized fluorescence stereomicroscope (Leica, Wetzlar, Germany). The embryos with the yolks removed were observed using the same method.

**Rescue experiments with JHA**

Methoprene (juvenile hormone analog [JHA], SDS Biotech, Tokyo, Japan) was kindly provided by Sho Sakurai (Kanazawa University). To examine the rescue effect of exogenous JH on early embryogenesis in the *Dri* null mutant of *B. mori*, we prepared a 0.02 μg/μL acetone solution of methoprene. We topically applied 250 nL of the methoprene solution or acetone alone (as a control) to the eggs derived from the sibling cross of *+/KO1*, either on day 0 or day 1 after oviposition. On day 3, we investigated the phenotypes of the embryos using PI staining and determined their genotypes through PCR analysis. In *P. hilaris*, 10 μL of methoprene solution (0.2 μg/μL) or acetone alone was topically applied to the dorsal abdomen of larvae immediately after *dsDri* injection, and the topical application was repeated every three days and immediately after molting. In *T. castaneum*, 125 nL of methoprene solution (0.2 μg/μL) or acetone alone was topically applied to the dorsal abdomen at L6D0 after *dsDri* injection at L4D0.

**RNAi experiments**

Template DNA fragments were PCR-amplified using the primers and templates listed in *SI* *Appendix*, Table S2 and Takara Ex Taq polymerase (Takara Bio) and purified using a Wizard SV Gel and PCR Clean-Up System (Promega). dsRNAs were synthesized from the amplified DNA using a RiboMAX T7 Large-Scale RNA Production System, following the manufacturer’s protocol (Promega). The concentrations of dsRNA were adjusted to 5 μg/μL, and approximately 2–4 μL and 40 nL of the dsRNA solution were injected into L2D0 and L3D0 larvae of *P. hilaris* and L4D0 larvae of *T. castaneum*, respectively. The phenotypes were observed using a binocular stereomicroscope daily until pupation or death.

**SI References**

1. A. M. Bolger, M. Lohse, B. Usadel, Trimmomatic: a flexible trimmer for Illumina sequence data. *Bioinformatics* **30**, 2114–2120 (2014).

2. M. Kawamoto, *et al.*, High-quality genome assembly of the silkworm, *Bombyx mori*. *Insect Biochem. Mol. Biol.* **107**, 53–62 (2019).

3. M. Pertea, D. Kim, G. M. Pertea, J. T. Leek, S. L. Salzberg, Transcript-level expression analysis of RNA-seq experiments with HISAT, StringTie and Ballgown. *Nat. Protoc.* **11**, 1650–1667 (2016).

4. H. Li, *et al.*, The Sequence Alignment/Map format and SAMtools. *Bioinformatics* **25**, 2078–2079 (2009).

5. B. Li, C. N. Dewey, RSEM: accurate transcript quantification from RNA-Seq data with or without a reference genome. *BMC Bioinformatics* **12**, 323 (2011).

6. M. G. Grabherr, *et al.*, Full-length transcriptome assembly from RNA-Seq data without a reference genome. *Nat. Biotechnol.* **29**, 644–652 (2011).

7. J. Sun, T. Nishiyama, K. Shimizu, K. Kadota, TCC: an R package for comparing tag count data with robust normalization strategies. *BMC Bioinformatics* **14**, 219 (2013).

8. K. J. Livak, T. D. Schmittgen, Analysis of relative gene expression data using real-time quantitative PCR and the 2(-ΔΔC(T)) Method. *Methods* **25**, 402–408 (2001).

9. S. Kuraku, C. M. Zmasek, O. Nishimura, K. Katoh, aLeaves facilitates on-demand exploration of metazoan gene family trees on MAFFT sequence alignment server with enhanced interactivity. *Nucleic Acids Res.* **41**, W22–W28 (2013).

10. K. Katoh, J. Rozewicki, K. D. Yamada, MAFFT online service: multiple sequence alignment, interactive sequence choice and visualization. *Brief. Bioinform.* **20**, 1160–1166 (2019).

11. S. Capella-Gutiérrez, J. M. Silla-Martínez, T. Gabaldón, trimAl: a tool for automated alignment trimming in large-scale phylogenetic analyses. *Bioinformatics* **25**, 1972–1973 (2009).

12. D. Darriba, *et al.*, ModelTest-NG: A new and scalable tool for the selection of DNA and protein evolutionary models. *Mol. Biol. Evol.* **37**, 291–294 (2020).

13. A. Stamatakis, RAxML version 8: a tool for phylogenetic analysis and post-analysis of large phylogenies. *Bioinformatics* **30**, 1312–1313 (2014).

14. I. Letunic, P. Bork, Interactive Tree Of Life (iTOL) v5: an online tool for phylogenetic tree display and annotation. *Nucleic Acids Res.* **49**, W293–W296 (2021).

15. Y. Naito, K. Hino, H. Bono, K. Ui-Tei, CRISPRdirect: software for designing CRISPR/Cas guide RNA with reduced off-target sites. *Bioinformatics* **31**, 1120–1123 (2015).

16. A. R. Bassett, C. Tibbit, C. P. Ponting, J.-L. Liu, Highly efficient targeted mutagenesis of *Drosophila* with the CRISPR/Cas9 system. *Cell Rep.* **4**, 220–228 (2013).

17. S. L. Gregory, R. D. Kortschak, B. Kalionis, R. Saint, Characterization of the dead ringer gene identifies a novel, highly conserved family of sequence-specific DNA-binding proteins. *Mol. Cell. Biol.* **16**, 792–799 (1996).

18. J. Huang, *et al.*, DPP-mediated TGFβ signaling regulates juvenile hormone biosynthesis by activating the expression of juvenile hormone acid methyltransferase. *Development* **138**, 2283–2291 (2011).

19. Y. Ishimaru, *et al.*, TGF-β signaling in insects regulates metamorphosis via juvenile hormone biosynthesis. *Proc. Natl. Acad. Sci. U. S. A.* **113**, 5634–5639 (2016).

**Figure, Table, and Dataset legends**

**Fig. S1.** Preliminary RNAi experiments using *Trilobium castaneum*. dsRNA of either *TBX20*, *Dfd*, or *Dri* was injected into *T. castaneum* on L4D0, and the occurrence of precocious metamorphosis was observed for 30 days (n = 6). The numbers represent the larval instar.

**Fig. S2.** Whole alignment of the predicted amino acid sequences of Dri in insects and vertebrates. Residues are color coded according to their conservancy. The regions of ARID follow the data obtained from previous studies (17). L.Bm, Lepidoptera *Bombyx mori*; L.Gm, Lepidoptera *Galleria mellonella*; D.Dm, Diptera *Drosophila melanogaster*; D.Hi, Diptera *Hermetia illucens*; D.Pp, Diptera *Phlebotomus papatasi*; Cole.Ph, Coleoptera *Psacothea hilaris*; Cole.Dv, Coleoptera *Diabrotica virgifera*; Cole.Nv, Coleoptera *Nicrophorus vespilloides*; Cole.Tc, Coleoptera *Tribolium castaneum*; N.Cc, Neuroptera *Chrysoperla carnea*; Hy.Mm, Hymenoptera *Microplitis mediator*; Hy.Am, Hymenoptera *Apis mellifera*; T.Tp, Thysanoptera *Thrips palmi*; B.Cs, Blattodea *Cryptotermes secundus*; He.Nl, Hemiptera *Nilaparvata lugens*; He.Hh, Hemiptera *Halyomorpha halys*; Or.Sa, Orthoptera *Schistocerca americana*; E.Nt, Ephemeroptera *Neocloeon triangulifer*; Od.Ie, Odonata *Ischnura elegans*; Coll.Fc, Collembola *Folsomia candida*; Ano.Dp, Anomopoda *Daphnia pulex*; I.Is, Ixodida *Ixodes scapularis*; Anu.Xt, Anura *Xenopus tropicalis*; R.Mm, Rodentia *Mus musculus*.

**Fig. S3.** Genome editing of *Dri* in *Bombyx mori.* (*A*) Schematic of the genomic structure of *Dri* in *B. mori*. Black arrow indicates the predicted transcription start site. Putative exons are boxed: UTRs and ORFs are white and shaded, respectively. Blue boxes and purple arrows are ARID and CRISPR-Cas9 target sites, respectively. (*B*) Knockout alleles of *Dri* generated in this study. Deletion regions are indicated by gray letters. Sky blue highlighting indicates the 5th exon of *Dri*. Blue and red highlighting show the regions of forward and reverse primers for genotyping, respectively. (*C*) Eggs obtained from the sibling cross of *Dri^+/KO1^* show two phenotypes: pigmentation and no pigmentation phenotypes. (*D*) Incidences of egg phenotype in *Dri^KO14^* and *Dri^KO15^* alleles. Eggs were obtained from the crosses of *+/+* × *+/+*, *+/+* × *+/KO*, or *+/KO* × *+/KO* in each allele. The data represent the frequency (%, n = 812 to 1360 eggs). Statistical analysis was carried out using Fisher’s exact test with the raw data, with Holm-adjusted *P* values (****P* < 0.001). (*E*) Genotypes of egg phenotypes were determined using PCR. *+/+* and *KO1/KO1* indicate each single band (858 and 260 bp, respectively), and *+/KO1* shows two heteroduplex bands (blue arrowheads, between 1000 and 1500 bp) in addition to two bands of 858 and 260 bp.

**Fig. S4.** RNAi experiments related to Fig. 3 in the main article. (*A*) Injection of *JHAMT* dsRNA on L2D0 induces precocious metamorphosis in *Psacothea hilaris*. The occurrence of precocious metamorphosis was observed in *P. hilaris* larvae injected with *JHAMT* dsRNA (n = 16). The numbers indicate the larval instar. (*B*) Effect of *Dri*-RNAi on the body weight and the size of the CA in *P. hilaris* larvae. Larvae were injected with dsRNAs of *EGFP* (negative control, n = 8) or *Dri* (N = 7) on L2D0, and the body weight and the diameter of CA were measured on L3D2. The data were analyzed using Student’s *t*-tests, and no significant differences were observed between the treatments (n.s., non-significant, *P* > 0.05).

**Fig. S5.** *Dri* RNAi induces precocious metamorphosis in *Trolobium castaneum*. (*A*) dsRNA of *MalE* (negative control) or *Dri* was injected into *T. castaneum* on L4D0, and precocious metamorphosis was observed (n = 11 or 17). The numbers represent the larval instar. (*B*) Phenotypes of *T. castaneum* injected with dsRNAs on L4D0. *dsMalE* (left) shows pupae metamorphosing from 7th instar larvae, and *Dri-*RNAi pupae (right) precociously metamorphosized from 6th instar larvae. Scale bar, 1 mm. (*C*) Rescue experiment with JH analog (JHA). JHA was topically applied on L6D0 after injection with *Dri* dsRNA on L4D0, and the inhibition of precocious metamorphosis was evaluated. Ctrl, acetone alone; JHA, methoprene.

**Fig. S6.** Relationship *Dri* and TGF-β signal in JH biosynthesis. (*A*) Schematic of JH biosynthesis regulated by TGF-β signal (18, 19). Enclosed P indicates the phosphorylation of Mad. (*B*–*D*) Expression levels of *Dri*, *JHAMT*, *DPP*, and *Mad* were examined in the CA derived from *Psacothea hilaris* larvae on day 4 after injection with *dsEGFP* (negative control, *B* to *D*), *dsDri* (*B*), *dsDPP* (*C*)*,* or *dsMad* (*D*) on day 0 of the 3rd instar. The transcript levels of *dsEGFP* were set as 100. The data represent the means ± SD (n = 3–5) and were analyzed using Student’s *t*-tests (****P* < 0.001; ***P* < 0.01; **P* < 0.05; not indicated, *P* > 0.05). (*E*) dsRNAs of *EGFP*, *Dri*, *Dpp*, or *Mad* were injected into *P. hilaris* on L2D0, and the phenotypes were observed (n = 8–18). D and P indicate death of larvae and pupation, respectively. (*F*) Phenotypes of 3rd- and 4th instar larvae injected with *dsEGFP* and *dsMad* in (*E*). Scale bar, 5 mm. (*G*) The larvae injected with dsRNAs were dissected along the left spiracles, and the digestive organs were removed from them. Light blue and pink boxes in (*G*) correspond to those of (*F*). Scale bar, 1 mm.

**Table S1.** Pheno- and genotypes of *Dri* null mutant embryos following JHA treatment

* Phenotypes 1 and 2 correspond to those presented in Fig. 2*D* in the main article.

**Table S2.** List of oligonucleotides used for PCR.

**Dataset S1.** Raw data and statistical results of figures.

**Dataset S2.** FASTA format file of Dri orthologs.

**Dataset S3.** Newick format file of phylogenetic tree.
