# Supplementary material for: Dead ringer acts as a major regulator of juvenile hormone biosynthesis in insects
Source: PNAS Nexus. 2024 Sep 30;3(10):pgae435. doi: 10.1093/pnasnexus/pgae435 (PMC11467689; doi:10.1093/pnasnexus/pgae435)
Supplement: pgae435_Supplementary_Data [file pgae435_supplementary_data.zip › Table S1.pdf]

Eggs treatment on	Treatment	Phenotype*	Genotype		
			+/+	+/*KO1*	*KO1*/*KO1*
Day 0	acetone	1	15	17	0
		2	0	0	9
	JHA	1	8	24	0
		2	0	0	16
Day 1	acetone	1	8	24	0
		2	0	0	16
	JHA	1	13	19	0
		2	0	0	16
