# Supplementary material for: Dead ringer acts as a major regulator of juvenile hormone biosynthesis in insects
Source: PNAS Nexus. 2024 Sep 30;3(10):pgae435. doi: 10.1093/pnasnexus/pgae435 (PMC11467689; doi:10.1093/pnasnexus/pgae435)
Supplement: pgae435_Supplementary_Data [file pgae435_supplementary_data.zip › Table S2.pdf]

Species	Primer name	Experiment	PCR template	Nucleotide sequence (5' to 3')
Bombyx mori	BmTF1 (dead ringer)_qPCR	qPCR	cDNA	GGTTTCCCATACGCACCACCTG<br>GATGACGAAGCAGAGCCCACTG
	BmTF2 (deformd)_qPCR	qPCR	cDNA	GCTCCTTCCTGATGAACGGCG<br>GATGTTGGTAGTATTCTCCTGGTGGTATG
	BmTF3 (TBX20)_qPCR	qPCR	cDNA	AGTGTTCGGATGGCGAGAGAGAC<br>CAAGATGTGGCTGGAGGAAGCG
	BmRp49_qPCR	qPCR	cDNA	CAGGCGGTTCAAGGGTCAATAC<br>TGCTGGGCTCTTTCCACGA
	BmDri_gRNA1	sgRNA syntheis	-	GAAATTAATACGACTCACTATAGGCAGTTGCACAAAAAAATTCGTTTTAGAGCTAGAAATAGC<br>AAAAGCACCGACTCGGTGCCACTTTTTCAAGTTGATAACGGACTAGCCTTATTTTAACTTGCTATTTCTAGCTCTAAAAC
	BmDri_gRNA2	sgRNA syntheis	-	GAAATTAATACGACTCACTATAGTGGGAATACTCGAATAAGTAGTTTTAGAGCTAGAAATAGC<br>AAAAGCACCGACTCGGTGCCACTTTTTCAAGTTGATAACGGACTAGCCTTATTTTAACTTGCTATTTCTAGCTCTAAAAC
	BmDri_gRNA3	sgRNA syntheis	-	GAAATTAATACGACTCACTATAGTATTATTAATAAGCTAAAACGTTTTAGAGCTAGAAATAGC<br>AAAAGCACCGACTCGGTGCCACTTTTTCAAGTTGATAACGGACTAGCCTTATTTTAACTTGCTATTTCTAGCTCTAAAAC
	BmDri_gRNA4	sgRNA syntheis	-	GAAATTAATACGACTCACTATAGTTCTAGTCTCGAAATGTTAGGTTTTAGAGCTAGAAATAGC<br>AAAAGCACCGACTCGGTGCCACTTTTTCAAGTTGATAACGGACTAGCCTTATTTTAACTTGCTATTTCTAGCTCTAAAAC
	BmDri_gRNA5	sgRNA syntheis	-	GAAATTAATACGACTCACTATAGTCTCGAAATGTTAGAGGCACGTTTTAGAGCTAGAAATAGC<br>AAAAGCACCGACTCGGTGCCACTTTTTCAAGTTGATAACGGACTAGCCTTATTTTAACTTGCTATTTCTAGCTCTAAAAC
	BmDri_gRNA6	sgRNA syntheis	-	GAAATTAATACGACTCACTATAGAGAGGCACAGGTTTATTTAAGTTTTAGAGCTAGAAATAGC<br>AAAAGCACCGACTCGGTGCCACTTTTTCAAGTTGATAACGGACTAGCCTTATTTTAACTTGCTATTTCTAGCTCTAAAAC
	BmDri_GT	genotyping	genomic DNA	CGCAGGGTTCTATACAGTTGTACCCATG<br>CGCGGCAGTTCCCGTGTACCACAC
Tribolium castaneum	TcTBX20_ds_1st	RNAi	cDNA	GAGCTCCGAACAGTTACTTGTCATTTAGAAACAAAG<br>GGCTGGTATTTGTGCATCGAATTCAGGAC
	TcTBX20_ds_2nd	RNAi	1st PCR product	GGATCCTAATACGACTCACTATAGGGAGCTCCGAACAGTTACTTGTC<br>GGATCCTAATACGACTCACTATAGGGGCTGGTATTTGTGCATCGAATTC
	TcDfd_ds_1st	RNAi	cDNA	GACGGCGTTACCGACGTATCAGCAG<br>GTCTCCTCATCGTCGTCGTCTTCGTC
	TcDfd_ds_2nd	RNAi	1st PCR product	GGATCCTAATACGACTCACTATAGGGACGGCGTTACCGACGTATC<br>GGATCCTAATACGACTCACTATAGGGTCTCCTCATCGTCGTCGTC
	TcDri_ds_1st	RNAi	cDNA	CCGATCAATCGACTGCCCATCATGGC<br>GCGAGTTGTTGCTGCTGGGAGACG
	TcDri_ds_2nd	RNAi	1st PCR product	GGATCCTAATACGACTCACTATAGGCCGATCAATCGACTGCCCATC<br>GGATCCTAATACGACTCACTATAGGGCGAGTTGTTGCTGCTGGGA
Escherichia coli	MalE_ds	RNAi	pMAL-c4E (NEB)	GGATCCTAATACGACTCACTATAGGTGATTGCTGCTGACGGGGGT<br>GGATCCTAATACGACTCACTATAGGTTTCTGGGCGTTTTCCATAGTGG
Drosophila melanogaster	DmDri_qPCR	qPCR	cDNA	CGAGTGCGAGAAAAAGAATCTGAGC<br>CGTCATCGGCATCTGGTTGTG
	DmRp49_qPCR	qPCR	cDNA	AAGCACTTCATCCGCCACCAG<br>TGTTGGGCATCAGATACTGTCCCT
Psacothea hilaris	PhDri_ds_1st	RNAi	cDNA	GGACATCAAGGTAGGGGACGACTTCG<br>CTCAATCGACGCTTCTCGCATTCGTAGG
	PhDri_ds_2nd	RNAi	1st PCR product	GGATCCTAATACGACTCACTATAGGGGACATCAAGGTAGGGGACG<br>GGATCCTAATACGACTCACTATAGGCTCAATCGACGCTTCTCGCATT
	PhDpp_ds_1st	RNAi	cDNA	GTGGATTGAGGATCCAAGGAGCAACCAC<br>GACGACTTTGTCCTCGTCGTCCAGATAC
	PhDpp_ds_2nd	RNAi	1st PCR product	GGATCCTAATACGACTCACTATAGGGTGGATTGAGGATCCAAGGAGC<br>GGATCCTAATACGACTCACTATAGGGACGACTTTGTCCTCGTCGTC
	PhMad_ds_1st	RNAi	cDNA	GTCCGACGGACGAAAGTAACTCAAACAC<br>GCTCATACGAATGGTGCACATTTTCGTGAG
	PhMad_ds_2nd	RNAi	1st PCR product	GGATCCTAATACGACTCACTATAGGGTCCGACGGACGAAAGTAACTC<br>GGATCCTAATACGACTCACTATAGGGCTCATACGAATGGTGCACATTTTC
	PhVvl_ds_1st	RNAi	cDNA	GAAGACGACACACCTACTAGCGACGAC<br>CGTCATTCGCTTCTCCTTTTGTCTCCTGTTG
	PhVvl_ds_2nd	RNAi	1st PCR product	GGATCCTAATACGACTCACTATAGGGAAGACGACACACCTACTAGCG<br>GGATCCTAATACGACTCACTATAGGCGTCATTCGCTTCTCCTTTTGTCT
	PhDri_qPCR	qPCR	cDNA	CGGGAAGTTCGCACTCGTCG<br>CCTCTTTGGGTCATCGTTTATCTCGTAG
	PhDpp_qPCR	qPCR	cDNA	CGATTGGACGACGAAAGAAGACC<br>CACCCTGGTTATGGCTCCGTG
	PhMad_qPCR	qPCR	cDNA	CAGGGTGACGAAGAGGAGAAATGG<br>GGGAATAGTTACGCACTTACTTGGAGTTC
	PhVvl_qPCR	qPCR	cDNA	CATCACCACCACCCCCATCAAG<br>CCCATACTTTGCTGTTGTTGTCTCTGG
	PhAACT_qPCR	qPCR	cDNA	GGTGGTGGTGGTGCTTCTTCG<br>CATAATAGCCCTTTTCCCGCTCAC
	PhHMGS_qPCR	qPCR	cDNA	GGACGTTTGGAAGTTGGAACTGAGAC<br>CCTTCAATGTCTGTCACACCGTAAGG
	PhHMGR_qPCR	qPCR	cDNA	GCCTACCATTACTTTGGATACCATCG<br>CGAAATAAGACAACATCTCAAGACGGTG
	PhMevK_qPCR	qPCR	cDNA	GGTCTTCACGGAAAACTTACAGGTGC<br>CGTTCTCCAAAACACTCGGGTCAG
	PhMevPK_qPCR	qPCR	cDNA	GCGAAAAACTCCAATCAATTTTAGGGG<br>GTTTCTGCGTAGCAACTCTTTAACGG
	PhMevPPD_qPCR	qPCR	cDNA	CATGCTGTCTGTTTAGATACTTTTCCTCC<br>GCCTGCGTCAAATGTATATGCAACC
	PhIPPI_qPCR	qPCR	cDNA	CGTGTTATTCTTCCAAGGGGACG<br>CAAAATCAACTGAAACCAAGGGGTC
	PhFPPS_qPCR	qPCR	cDNA	CAGTCCTTACCATTGCTATGTACCGATG<br>CCTTGATTTTTTATGATACCATCCAGGGAC
	PhJHAMT_qPCR	qPCR	cDNA	GGCTAGTAATCCCATCTACGAGGTGTACC<br>CAGGTTCCTTCAGACCGTGGTAGG
	PhRp49_qPCR	qPCR	cDNA	CATTAGGCATCAATCAGACAGGTATTCC<br>CGAGTCTTTGAGTTTGAACCATAACCAATG
Aequorea victorea	EGFP_ds	RNAi	pEGFP (Clontech)	GGATCCTAATACGACTCACTATAGGNATGGTGAGCAAGGGCGAGGA<br>GGATCCTAATACGACTCACTATAGGNTTACTTGTACAGCTCGTCCA
