# Supplementary figures and images for: Dead ringer acts as a major regulator of juvenile hormone biosynthesis in insects
Source: PNAS Nexus. 2024 Sep 30;3(10):pgae435. doi: 10.1093/pnasnexus/pgae435 (PMC11467689; doi:10.1093/pnasnexus/pgae435)

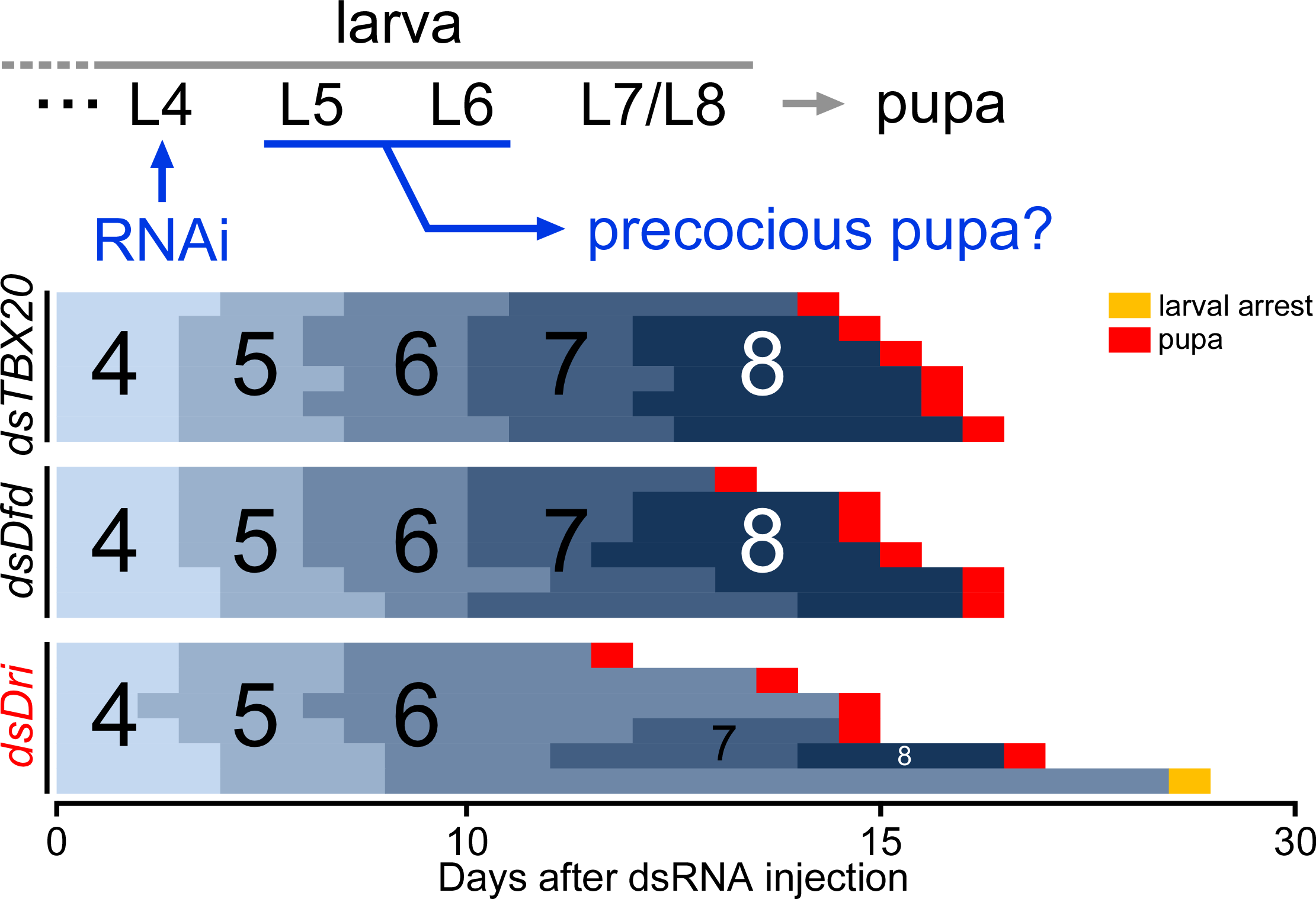

Supplement: pgae435_Supplementary_Data [file pgae435_supplementary_data.zip › Figure S1.tif]

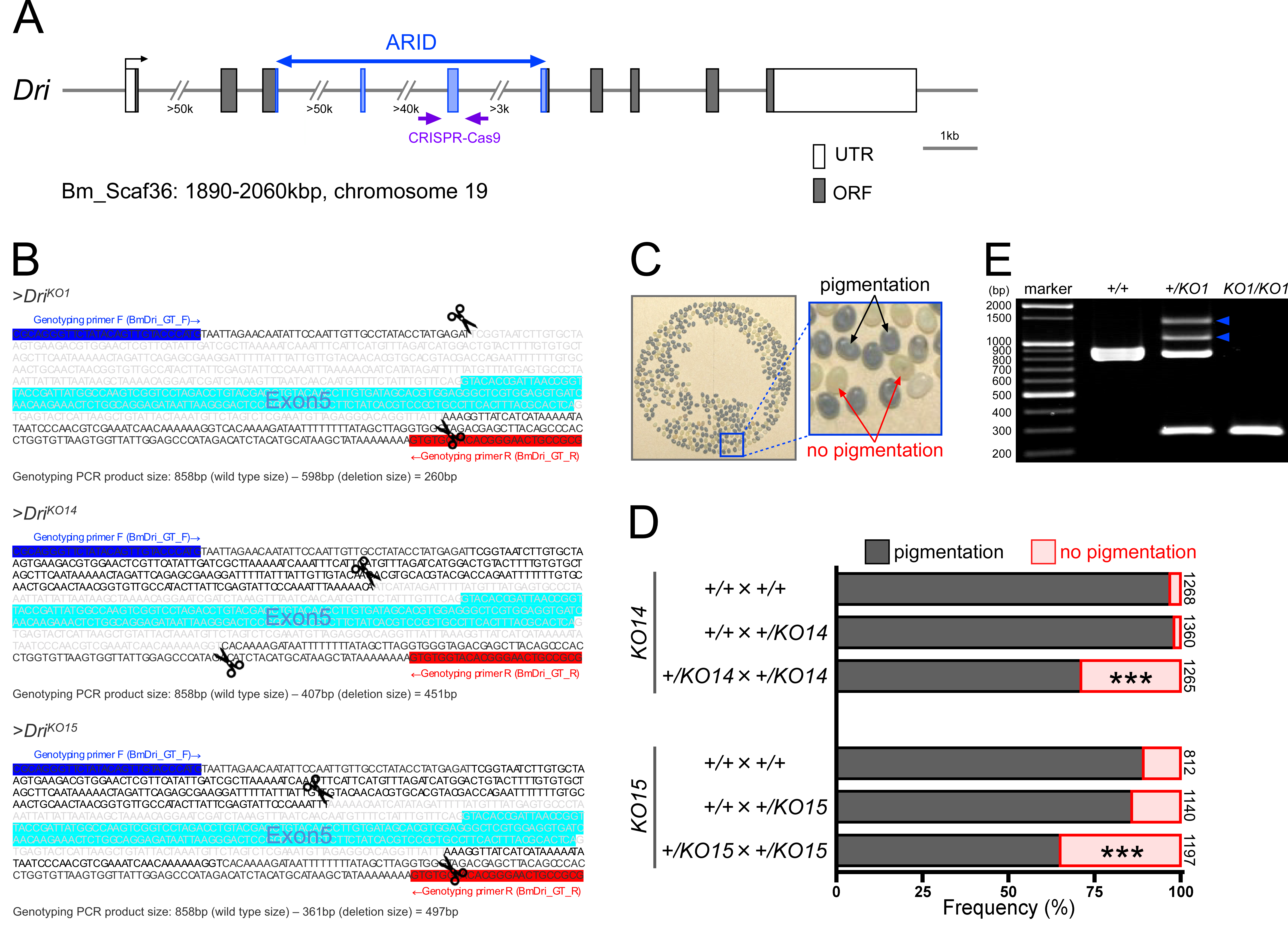

Supplement: pgae435_Supplementary_Data [file pgae435_supplementary_data.zip › Figure S3.tif]

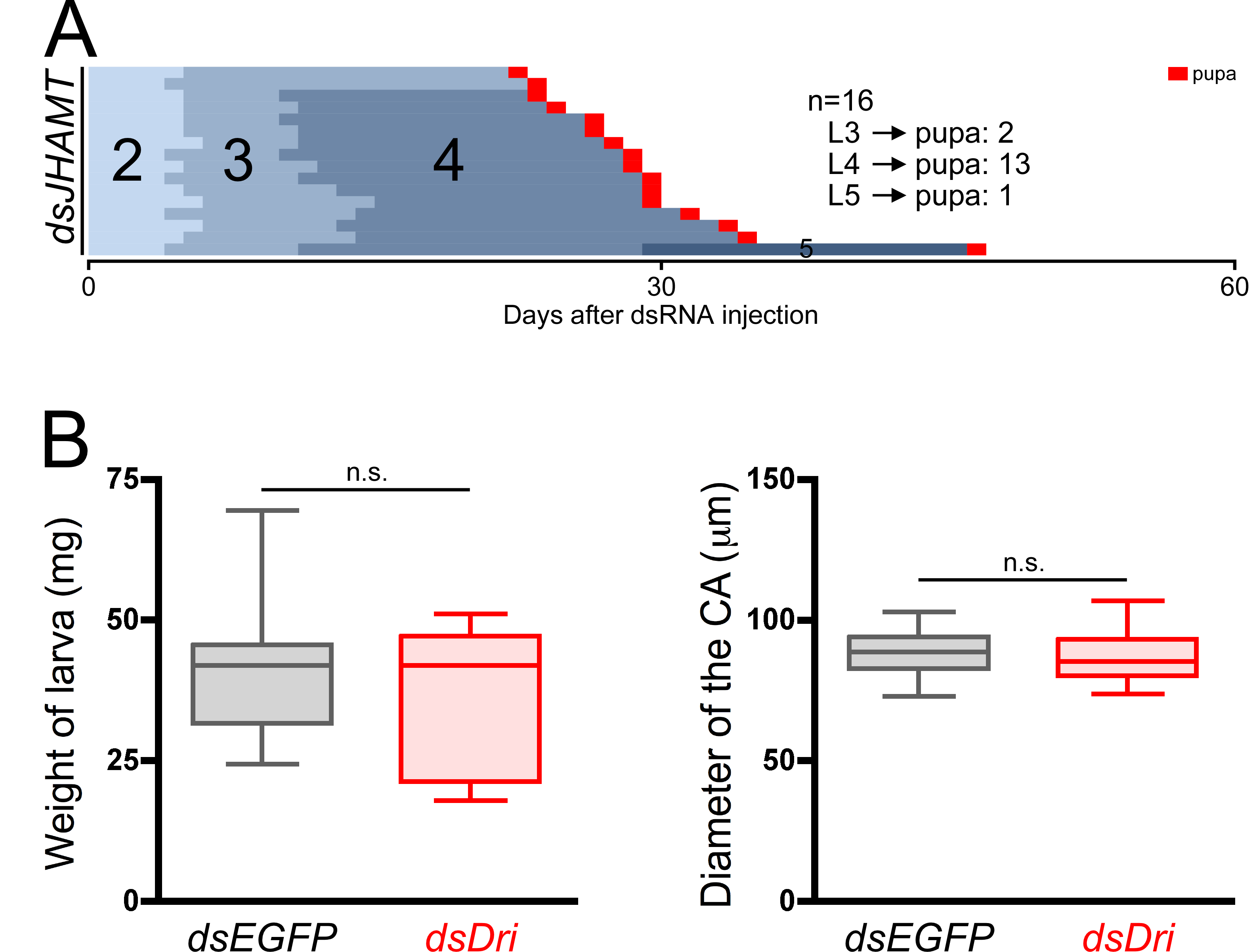

Supplement: pgae435_Supplementary_Data [file pgae435_supplementary_data.zip › Figure S4.tif]

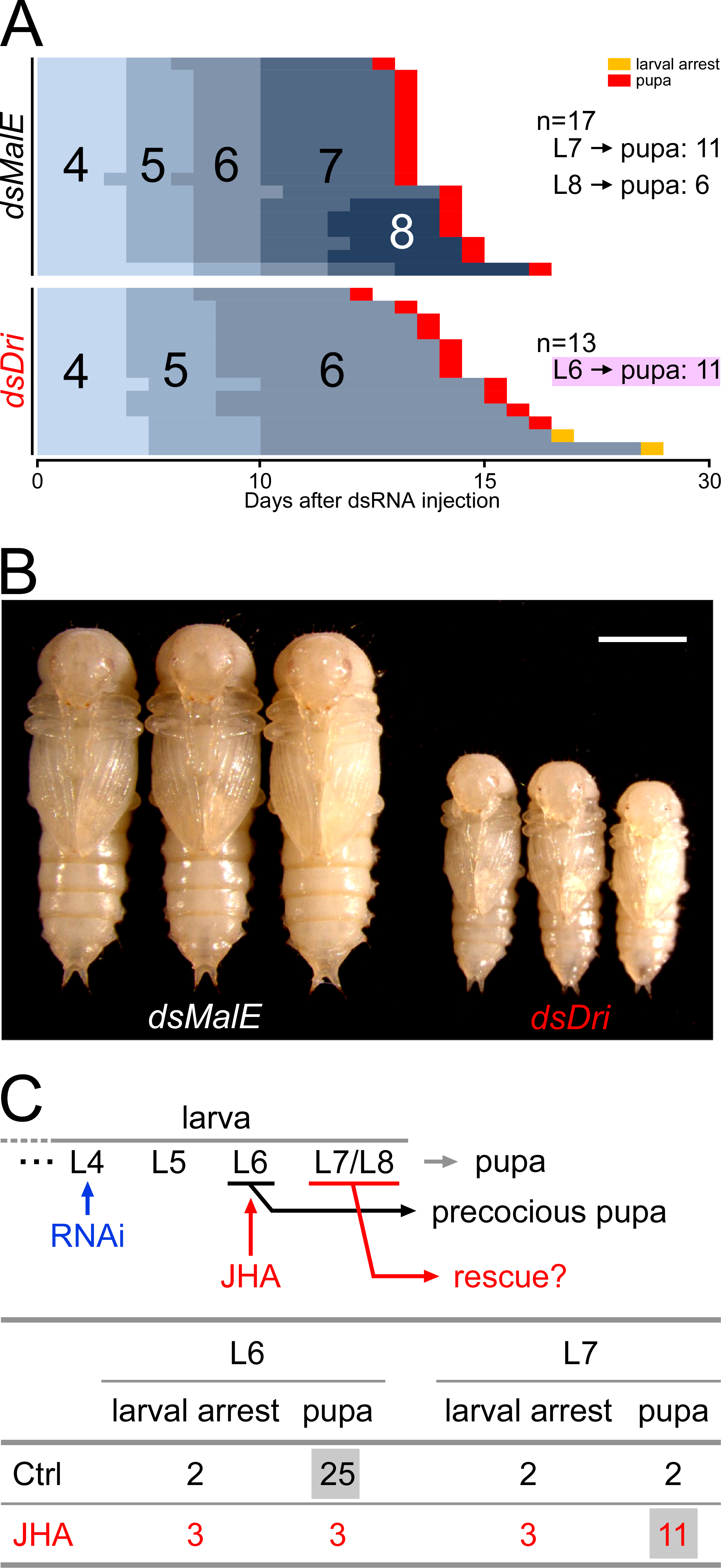

Supplement: pgae435_Supplementary_Data [file pgae435_supplementary_data.zip › Figure S5.tif]

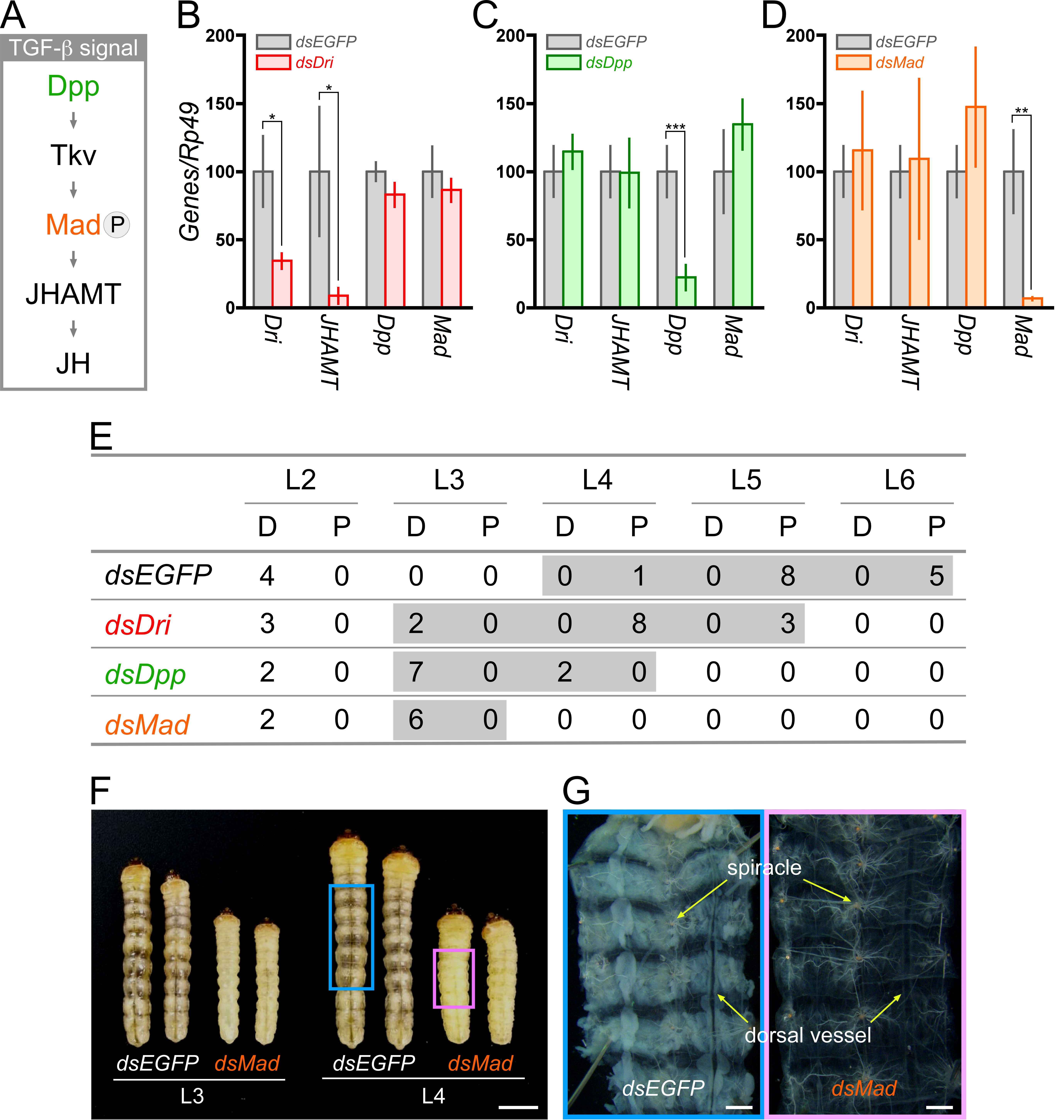

Supplement: pgae435_Supplementary_Data [file pgae435_supplementary_data.zip › Figure S6.tif]
